# Association between body fat parameters and arterial stiffness

**DOI:** 10.1038/s41598-021-00175-z

**Published:** 2021-10-15

**Authors:** Hack-Lyoung Kim, Dong-Won Ahn, Su Hwan Kim, Dong Seok Lee, Soon Ho Yoon, Joo-Hee Zo, Myung-A. Kim, Ji Bong Jeong

**Affiliations:** 1 Division of Cardiology, Department of Internal Medicine, Boramae Medical Center, Seoul National University College of Medicine, Seoul, South Korea; 2 Division of Gastroenterology and Hepatology, Department of Internal Medicine, Boramae Medical Center, Seoul National University College of Medicine, 20 Borame-ro 5-gil, Dongjak-gu, Seoul, 07061 South Korea; 3 Department of Radiology, Seoul National University College of Medicine, Seoul National University Hospital, Seoul, South Korea

**Keywords:** Cardiology, Medical research, Risk factors

## Abstract

The influence of body fat on arterial stiffness remains controversial. This study was performed to investigate the associations between four different types of body fat parameters and brachial-ankle pulse wave velocity (baPWV). A total of 3758 subjects (mean age, 53.4 ± 8.8 years; females, 36.3%) who underwent health check-up were retrospectively analyzed. Anthropometric parameters including body mass index (BMI), waist circumference (WC) and waist–hip ratio (WHR) were assessed, and visceral fat area (VFA) was calculated by bioelectrical impedance analysis. In simple linear correlation analyses, baPWV was associated with WC, WHR and VFA (*P* < 0.001 for each), but not with BMI (*P* = 0.175). In multivariable analyses, BMI and WC were not associated with baPWV (*P* > 0.05 for each). Even after controlling for potential confounders, higher baPWV was significantly associated not only with higher WHR [for > 0.90 in men and > 0.85 in women: odds ratio (OR), 1.23; 95% confidence interval (CI), 1.06–1.42; *P* = 0.005; for the highest tertile compared to the lowest tertile: OR, 1.38; 95% CI, 1.15–1.66; *P* < 0.001], but also with higher VFA (for ≥ 100 cm^2^: OR, 1.39; 95% CI, 1.20–1.60; *P* < 0.001; for the highest tertile compared to the lowest tertile: OR, 1.77; 95% CI, 1.48–2.12; *P* < 0.001). Our study showed that baPWV was correlated with WHR and VFA, but not with BMI and WC. This implies that arterial stiffness may be more strongly associated with abdominal obesity than overall obesity.

## Introduction

Arterial wall gradually stiffened due to aging and prolonged exposure to various stressful conditions such as high blood pressure, hyperglycemia, dyslipidemia, smoking and inflammation^1,2^. Although there are various methods to measure arterial stiffness, pulse wave velocity is the most widely used because of its non-invasiveness, simplicity and rich clinical data^3^. Of clinical significance, the information on arterial stiffness has predicted future cardiovascular events in a variety of patients as well as the general population independent of traditional risk factors^4–6^. Therefore, it is important to find factors related to arterial stiffness because it can be applied to cardiovascular prevention and treatment strategies.

As the number of obese people around the world is steadily increasing and the morbidities related to obesity have become a big problem in human society^7,8^. Anthropometric parameters and other indicators related to obesity and body fat in our bodies are receiving increasing attention. Given that the association between obesity and cardiovascular disease is well established^9,10^, vascular dysfunction has been suggested as one of the factors linking these two pathological states^11^. However, the relationship between arterial stiffness and adiposity is still inconsistent^12–15^. It is also known that cardiovascular risk varies depending on the location of body fat in our body: visceral fat is more strongly associated with cardiovascular risk than subcutaneous fat^16–18^. Considering this, it would be valuable to examine the relationships between measures of arterial stiffness and obesity according to the location of body fat.

In this study, we investigated the associations of brachial-ankle PWV (baPWV) with several parameters of body fat including body mass index (BMI), waist circumference (WC), waist–hip ratio (WHR) and visceral fat area (VFA). Our hypothesis was that indicators of abdominal fat including WC, WHR and VFA, were more significantly associated with baPWV than that an indicator of overall obesity, BMI.

## Methods

### Study population

**Figure 1:**
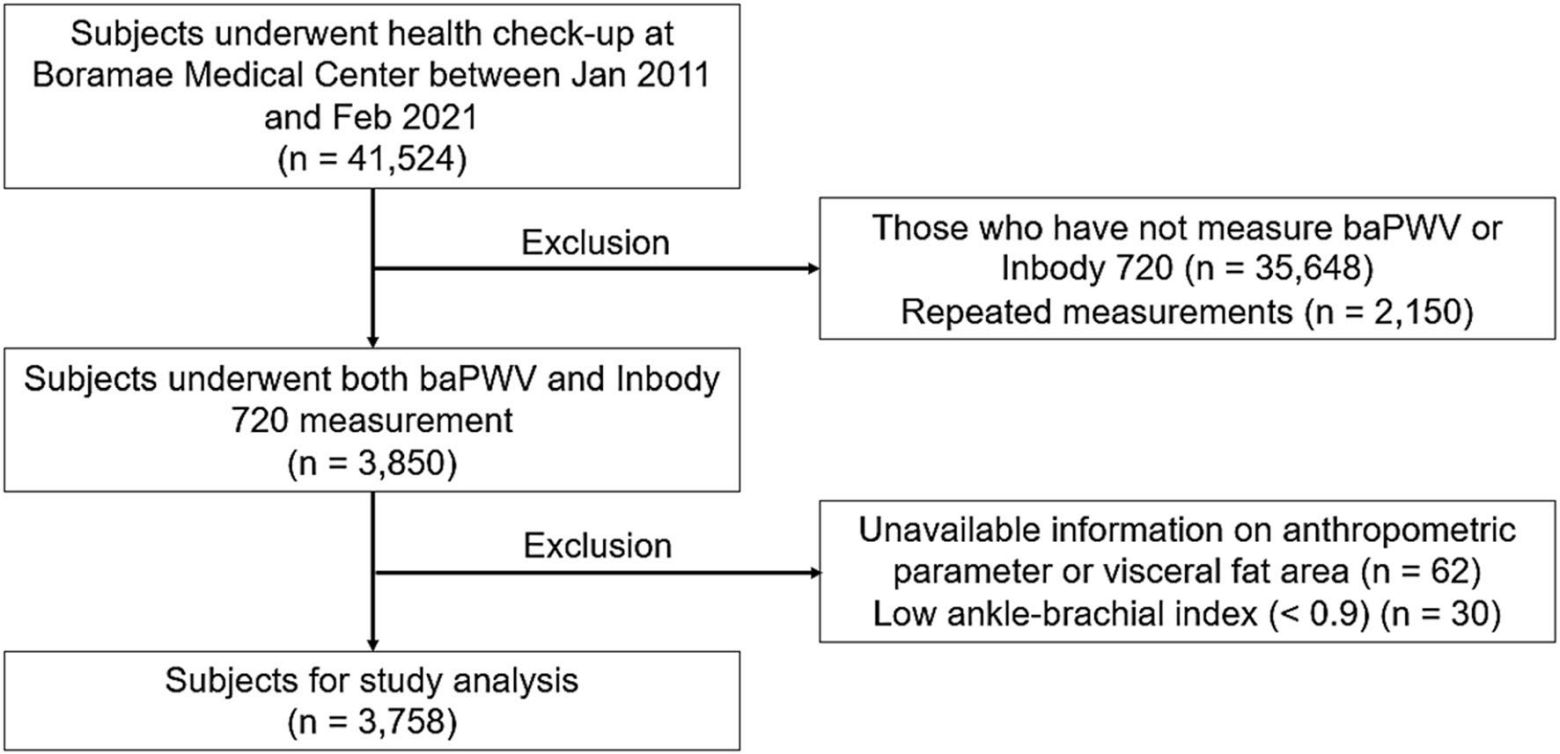
Flow chart of enrollment of study subjects. *baPWV* brachial-ankle pulse wave velocity.

This single center and the retrospective study was performed at a general hospital in a big city (Seoul, South Korea). Between January 2011 and February 2021, both baPWV and Inbody 720 measurements were made in 3850 of 41,524 subjects who underwent voluntary health check-up at the health care center of Boramae Medical Center (Seoul, South Korea). After exclusion of 92 subjects with unavailable information on anthropometric parameter of VFA (n = 62), and low ankle-brachial index (< 0.9) (n = 30), 3758 subjects were finally analyzed in this study. The study flow of subject enrollment is demonstrated in Fig. 1. This study conforms to the ethical guidelines of the Declaration of Helsinki, and the study protocol was approved by the Institutional Review Board (IRB) of Boramae Medical Center (Seoul, Korea) (IRB number, 30-2021-95). Obtaining informed consent was waived by the IRB of Boramae Medical Center (Seoul, Korea) due to retrospective study design and routine nature of data collected.

### Clinical data

Blood pressure was measured on the right upper arm using an oscillometric device by a trained nurse. Hypertension was defined as systolic blood pressure ≥ 140 mmHg, diastolic blood pressure ≥ 90 mmHg, or the use of anti-hypertensive medications. Diabetes mellitus was defined as fasting plasma glucose ≥ 126 mg/dL, glycated hemoglobin ≥ 6.5%, or anti-diabetic medications. Dyslipidemia was defined as triglyceride ≥ 150 mg/dL, high-density lipoprotein < 40 mg/dL in men and < 50 mg/dL in women, or the use of anti-dyslipidemic medications. Information on cigarette smoking, alcohol drinking, and previous history of coronary artery disease and stroke was obtained based on the questionnaires. After overnight fasting for about 12 h, blood levels of the following parameters were obtained using commercially available chemistry analyzer (Modular DP and Cobas^®^ 8000, Roche. Diagnostics, Mannheim, Germany; G11vr, Tosoh Bioscience, Inc., Tokyo, Japan): glucose, glycated hemoglobin, total cholesterol, low-density lipoprotein cholesterol, high-density lipoprotein cholesterol, triglyceride, aspartate transaminase, alanine aminotransferase, gamma-glutamyl transferase and C-reactive protein.

### Anthropometric data

At the day of health check-up height and body weight were measured. BMI was calculated as body weight divided by height squared (kg/m^2^). Normal weight, overweight and obesity were defined as BMI < 23 kg/m^2^, 23 ~ 24.9 kg/m^2^, and ≥ 25 kg/m^2^, respectively^19^. WC was measured by a trained nurse while exhaling in a standing position just above the hip bone with a tape measure. During the measurement, the tape was hold flat against body, not too tight and take a reading. Abdominal obesity was defined as WC ≥ 90 cm in men and ≥ 85 cm in women^19^. Hip circumference was measured around the widest portion of the buttocks, with the tape parallel to the floor. WHR was calculated as WC/hip circumference. Abdominal obesity was defined as WHR > 0.90 cm in men and > 0.85 cm in women^20^.

### VFA

VFA was automatically calculated by bioelectrical impedance analysis using Inbody 720 (Biospace Co., Seoul, South Korea)^21^. It has been reported that VFA measurements using the Inbody 720 well correlated with the results of computed tomography (CT)^22^. In our health screening cohort, there was 2441 subjects underwent CT examination. Pearson’s bivariate correlation analysis showed a significant and strong correlation between VFA measured by INbody 720 and by CT (*r* = 0.718, *P* < 0.001) (Supplementary Figure S1). A subject with VFA ≥ 100 cm^2^ was considered to have visceral obesity^23^.

### baPWV

The baPWV was automatically generated using a VP-1000 analyzer (Collin Co., Komaki, Japan)^24,25^. Measurement of baPWV was performed in an isolated room with constant temperature and humidity in the morning. All subjects were fasting, taking no cardiovascular medications, and resting in a supine position for at least five minutes prior to measurement. Blood pressure cuffs were wrapped around the upper arms and both ankles, and pressure wave forms of the brachial and tibial arteries were recorded with plethysmographic and oscillometric pressure sensors. Time differences between the points where the pulse waveform starts to rise in the systolic phase in the brachial and tibial arteries were measured. The distance between the two points was automatically calculated from the subject’s height, and baPWV was calculated by dividing the distance between the two points by the time difference. The average value of right and left baPWV was used in our study analysis. Measurement of baPWV was performed by a single experienced expert. The coefficient of variation in baPWV measurement for intra-observer variability was 5.1% in our laboratory^26^.

### Statistical analysis

Continuous variables are expressed as mean ± standard deviation, and categorical variables are expressed as n (%). Study subjects were stratified into two groups according to the median value of baPWV: subjects with higher baPWV (≥ 1372 cm/s) and those with lower baPWV (< 1372 cm/s). Continuous variables were compared using Student’s *t*-test, and categorical variables were compared using the *χ*^2^ test between the two groups. The simple linear correlation between two continuous variables was analyzed using Pearson’s bivariate correlation analysis, and demonstrated using scatter plot. Binary multiple logistic regression analysis was performed to find independent associations of anthropometric measures and VFA with baPWV. The following clinical covariates were adjusted in this multivariable analysis: age, sex, hypertension, diabetes mellitus, dyslipidemia, cigarette smoking and alcohol drinking. Multiple linear regression analysis was also performed to show independent associations between body fat parameters and baPWV after controlling for age and sex. *P* value of < 0.05 was considered statistically significant. All statistical analyses were performed using SPSS 22.0 (IBM Corp., Armonk, NY, USA).

## Results

**Figure 2:**
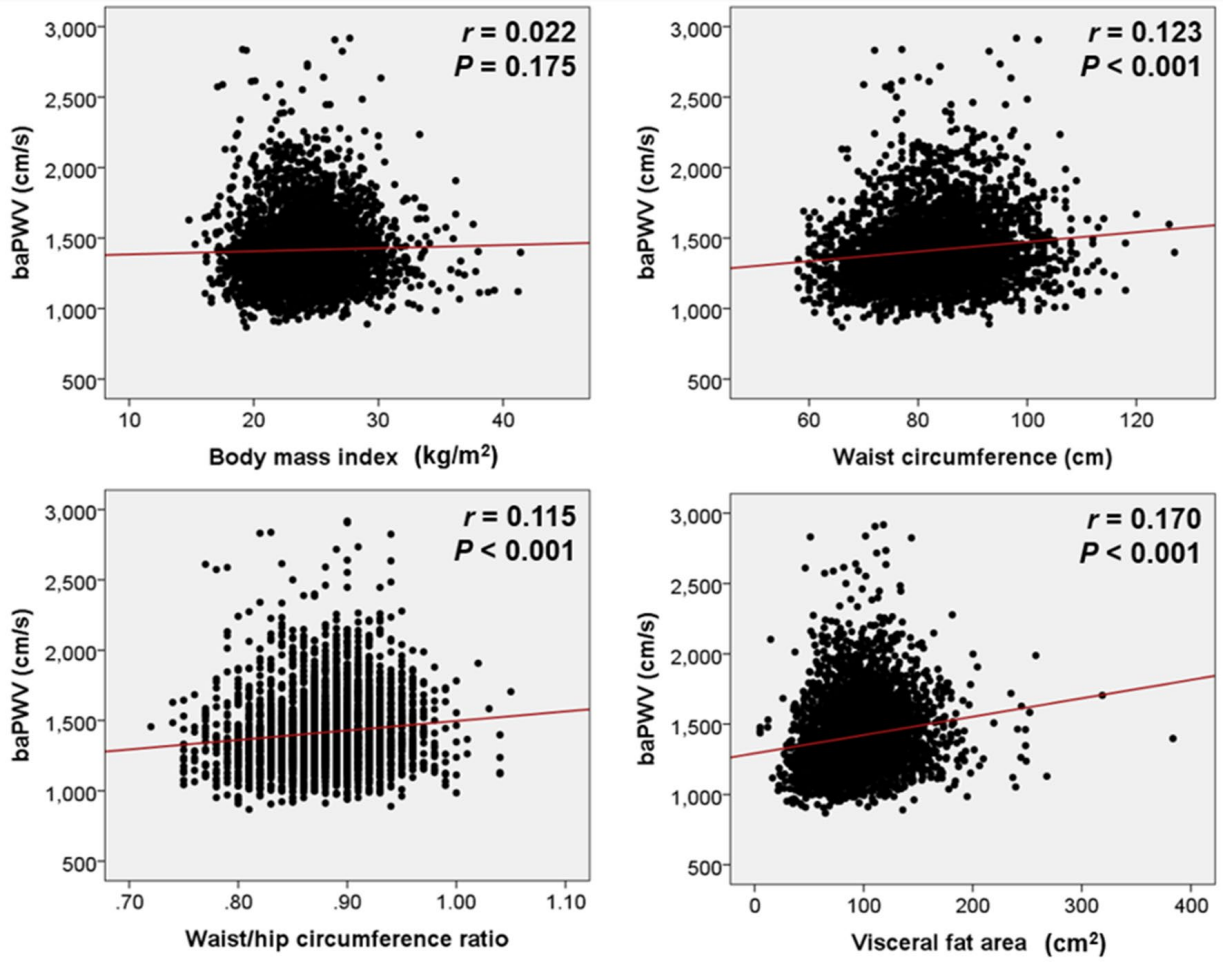
Scatter plots showing correlations of baPWV with four body fat parameters. *baPWV* brachial-ankle pulse wave velocity.

The clinical characteristics of 3,758 total study subjects and comparisons of clinical characteristics between subjects with higher (≥ 1372 cm/s) and lower (< 1372 cm/s) baPWV are demonstrated in Table 1. In total subjects, mean age was 53.4 ± 8.8 years and male predominant (63.7%). The prevalence of hypertension, diabetes mellitus and dyslipidemia, and previous history of coronary artery disease and stroke were 21.9%, 8.6%, 15.5%, 1.3%, and 0.4%, respectively. The results of main laboratory tests were within normal limits. Subjects with higher baPWV (≥ 1372 cm/s) were older and had higher blood pressure than those with lower baPWV (< 1372 cm/s). Cardiovascular risk factors including hypertension, diabetes mellitus, dyslipidemia and previous coronary artery disease were more prevalent in subjects with higher baPWV than those with lower baPWV. The results of laboratory tests were unfavorably presented in subjects with higher baPWV than those with lower baPWV. The parameters of body fat are shown in Table 2. Although there was a somewhat difference according to the parameters, about one third (27.5–41.1%) were obese. All 4 body fat parameters including BMI, WC, WHR and VFA were significantly higher in subjects with higher baPWV than those with lower baPWV. In simple linear correlation analyses, baPWV was associated with WC, WHR and VFA (*P* < 0.001 for each), but not with BMI (*P* = 0.175) (Table 3). These associations are demonstrated as scatter plots in Fig. 2. In multivariable binary logistic regression analyses, BMI and WC were not associated with baPWV (*P* > 0.05 for each). Even after controlling for potential confounders, higher baPWV was significantly associated with not only with higher WHR (for > 0.90 in men and > 0.85 in women: odds ratio [OR], 1.23; 95% confidence interval [CI], 1.06–1.42; *P* = 0.005; for the highest tertile compared to the lowest tertile: OR, 1.38; 95% CI, 1.15–1.66; *P* < 0.001), but also with higher VFA (for ≥ 100 cm^2^: OR, 1.39; 95% CI, 1.20–1.60; *P* < 0.001; for the highest tertile compared to the lowest tertile: OR, 1.77; 95% CI, 1.48–2.12; *P* < 0.001) were significantly associated with higher baPWV (Table 4).

**Table 1:** Clinical characteristics of study subjects according to baPWV.

Characteristic	Total subjects (n = 3758)	Subjects with baPWV ≥ 1372 cm/s (n = 1885)	Subjects with baPWV < 1372 cm/s (n = 1873)	*P*
Age, years	53.4 ± 8.8	56.6 ± 8.1	50.2 ± 8.3	< 0.001
Female sex	1,363 (36.3)	608 (32.3)	755 (40.3)	< 0.001
Systolic BP, mmHg	123 ± 14	130 ± 13	115 ± 10	< 0.001
Diastolic BP, mmHg	76.8 ± 10.4	81.8 ± 9.5	71.9 ± 8.8	< 0.001
**Cardiovascular risk factors**				
Hypertension	822 (21.9)	589 (31.2)	233 (12.4)	< 0.001
Diabetes mellitus	324 (8.6)	253 (13.4)	71 (3.8)	< 0.001
Dyslipidemia	582 (15.5)	378 (20.1)	204 (10.9)	< 0.001
Cigarette smoking	480 (12.8)	229 (12.1)	251 (13.4)	0.250
Alcohol drinking	702 (18.7)	366 (19.4)	336 (17.9)	0.245
Previous CAD	50 (1.3)	38 (2.0)	12 (0.6)	< 0.001
Previous stroke	14 (0.4)	10 (0.5)	4 (0.2)	0.111
**Laboratory findings**				
Fasting glucose, mg/dL	97.2 ± 21.1	101.7 ± 24.7	92.6 ± 15.3	< 0.001
Glycated hemoglobin, %	5.76 ± 0.76	5.9 ± 0.9	5.5 ± 0.4	< 0.001
Total cholesterol, mg/dL	195 ± 36	195 ± 38	195 ± 33	0.965
LDL cholesterol, mg/dL	119 ± 33	119 ± 36	119 ± 31	0.998
HDL cholesterol, mg/dL	54.3 ± 13.9	53.1 ± 13.3	55.6 ± 14.4	< 0.001
Triglyceride, mg/dL	113 ± 71	120 ± 73	106 ± 67	< 0.001
AST, IU/L	27.8 ± 12.7	29.1 ± 13.5	26.4 ± 11.7	< 0.001
ALT, IU/L	27.9 ± 18.5	29.5 ± 13.5	26.2 ± 17.4	< 0.001
GGT, IU/L	33.4 ± 44.8	38.2 ± 56.5	28.7 ± 27.8	< 0.001
C-reactive protein, mg/L	1.45 ± 4.67	0.16 ± 0.59	0.12 ± 0.28	0.010

Numbers are expressed as mean ± standard deviation or n (%).

*baPWV* brachial-ankle pulse wave velocity, *BP* blood pressure, *CAD* coronary artery disease, *LDL* low-density lipoprotein, *HDL* high-density lipoprotein, *AST* aspartate transaminase, *ALT* alanine aminotransferase, *GGT* gamma-glutamyl transferase.

**Table 2:** Body fat parameters according to baPWV.

Parameter	Total subjects (n = 3758)	Subjects with baPWV ≥ 1372 cm/s (n = 1885)	Subjects with baPWV < 1372 cm/s (n = 1873)	*P*
Body mass index, kg/m^2^	24.0 ± 3.1	24.1 ± 2.9	23.9 ± 3.2	0.048
Body mass index ≥ 25 kg/m^2^	1369 (36.4)	710 (37.7)	659 (35.2)	0.114
Waist circumference, cm	83.1 ± 9.3	84.0 ± 8.9	82.1 ± 9.5	< 0.001
Waist circumference, men ≥ 90 cm, women ≥ 85 cm	1035 (27.5)	567 (30.1)	468 (25.0)	< 0.001
Waist–hip ratio	0.87 ± 0.04	0.88 ± 0.04	0.87 ± 0.04	< 0.001
Waist–hip ratio, men > 0.90, women > 0.85	1545 (41.1)	836 (44.4)	709 (37.9)	< 0.001
Visceral fat area, cm^2^	94.2 ± 32.0	99.2 ± 31.6	89.1 ± 31.5	< 0.001
Visceral fat area ≥ 100 cm^2^	1466 (39.0)	858 (45.5)	608 (32.5)	< 0.001

*baPWV* brachial-ankle pulse wave velocity.

**Table 3:** Simple linear correlations showing the associations between body fat parameters and baPWV.

Parameter	*r*	*P*
Body mass index	0.022	0.175
Waist circumference	0.123	< 0.001
Waist-hip ratio	0.115	< 0.001
Visceral fat area	0.170	< 0.001

*baPWV* brachial-ankle pulse wave velocity.

**Table 4:** Multiple binary logistic regression analyses showing independent associations of body fat parameters with higher baPWV (≥ 1372 cm/s).

Parameter	OR (95% CI)	*P*
Body mass index ≥ 25 kg/m^2^	0.86 (0.74–1.00)	0.051
Body mass index < 23 kg/m^2^	1	
Body mass index, 23–24.9 kg/m^2^	1.11 (0.93–1.33)	0.209
Body mass index ≥ 25 kg/m^2^	0.91 (0.77–1.07)	0.271
Body mass index, the lowest tertile	1	
Body mass index, middle tertile	1.09 (0.92–1.29)	0.276
Body mass index, the highest tertile	0.86 (0.72–1.03)	0.113
Waist circumference, men ≥ 90 cm, women ≥ 85 cm	1.02 (0.87–1.19)	0.756
Waist circumference, the lowest tertile	1	
Waist circumference, middle tertile	1.26 (1.06–1.50)	0.007
Waist circumference, the highest tertile	1.10 (0.91–1.32)	0.307
Waist-hip ratio, men > 0.90, women > 0.85	1.23 (1.06–1.42)	0.005
Waist-hip ratio, the lowest tertile	1	
Waist-hip ratio, middle tertile	1.43 (1.21–1.68)	< 0.001
Waist-hip ratio, the highest tertile	1.38 (1.15–1.66)	< 0.001
Visceral fat area ≥ 100 cm^2^	1.39 (1.20–1.60)	< 0.001
Visceral fat area, the lowest tertile	1	
Visceral fat area, middle tertile	1.52 (1.28–1.80)	< 0.001
Visceral fat area, the highest tertile	1.77 (1.48–2.12)	< 0.001

Following clinical covariates are controlled: age, sex, hypertension, diabetes mellitus, dyslipidemia, cigarette smoking and alcohol drinking.

*baPWV* brachial-ankle pulse wave velocity, *OR* odds ratio, *CI* confidence interval.

In multiple linear regression analyses, baPWV was associated with WC (*β* = 0.085; *P* < 0.001), WHR (*β* = 0.081; *P* < 0.001) and VFA (*β* = 0.116; *P* < 0.001) but not with BMI (P = 0.412) even after controlling for age and sex (Supplementary Table S1). In age-specific analysis (Supplementary Table S2), WHR and VFA were more strongly associated with baPWV in young age group (< 55 years) than in those older age group (≥ 55 years). Both BMI and WC were not associated with baPWV in both age groups.

## Discussion

Our study showed that baPWV correlated more strongly with WHR and VFA than with BMI and WC. BMI was not correlated with baPWV even in simple correlation analysis. Although WC was positively correlated with baPWV in univariable analysis, its significance disappeared after controlling for potential confounders in multivariable logistic regression analysis. The correlations of baPWV with WHR and VFA remained even after controlling for important clinical covariates. The correlation between baPWV and VFA was strongest. Given that the degree of abdominal obesity is better expressed by WC, WHR and VFA than by BMI, these results suggest that abdominal obesity is more strongly associated with arterial stiffness than overall obesity.

### Previous studies on the association between body fat parameters and arterial stiffness

There are several studies that looked at the association between body fat parameters and arterial stiffness^12–15,27–31^. We summarized the results of these studies (Supplementary Table S3). Their results are inconsistent and do not conclusively establish an association of arterial stiffness with body fat parameters. In addition, body fat parameters used in most of these studies are anthropometric indices^12–14,27,28,31^. The use of a more objective indicator, VFA, has been used in only a few studies^15,29,30^, but the number of patients analyzed in these is relatively small (50–344 subjects)^15,29,30^. On the other hand, in our study, we used VFA as well as anthropometric indices, and the number of study subjects was very large with 3758 subjects. In addition, although it is relatively well-known that the location of fat in our body has different effects on the cardiovascular system, there are few studies that analyzed the association between measures of arterial stiffness and obesity according to the location of body fat. We used both overall (BMI) and visceral obesity (WC, WHR and VFA) indices for analysis, and suggested that the visceral obesity index is more correlated with arterial stiffness than overall obesity index.

Although not focused on the association between obesity indices and arterial stiffness according to the location of body fat, a few studies have addressed this issue^27–30^. In a study of 146 middle-aged adults, both carotid-femoral pulse wave velocity (cfPWV) and baPWV had stronger associations with WC and VFA than with BMI^27^. A study that examined 10,197 Chinese subjects who underwent health check-up showed that baPWV was more strongly correlated with WHR than BMI and WC^28^. In a study of 344 patients who underwent kidney transplantation, WHR and VFA were significantly associated with baPWV and cfPWV in univariable analysis; however, the association disappeared in multivariable analysis^29^. Sex difference was shown in some studies. Nordstrand and colleagues have shown that increasing BMI, WC, WHR and VFA were independently associated with higher cfPWV in women but not in men among 133 morbidly obese patients^30^. Similar findings were observed in another study of 2647 healthy individuals demonstrating that baPWV correlated with BMI and WC only in women but not in men^31^. All of these studies, including ours indicate that abdominal obesity is more strongly associated with arterial stiffness than overall obesity.

### Pathophysiology

Abdominal obesity is more harmful to cardiovascular system than overall obesity^17^. Visceral fat secretes a variety of cytokines, leading to chronic inflammatory conditions, endothelial cell dysfunction and insulin resistance^17,32,33^. These unfavorable factors may also contribute to vascular pathology such as increased arterial stiffness^27,34^. In particular, increased circulating leptin level elevates blood pressure and sympathetic tone, leading to arterial stiffening^35^. Also, shared traditional cardiovascular risk factors such as high blood pressure, hyperglycemia, dyslipidemia and cigarette smoking can explain the association between abdominal obesity and arterial stiffening^36^. It is inferred that increased arterial stiffness may be at least partially responsible for poor cardiovascular prognosis in patients with abdominal obesity. Also, the opposite hypothesis is possible: increased cardiovascular risk in patients with increased arterial stiffness may be attributable in part to abdominal obesity.

### Clinical implications

For physicians treating obesity, improving poor cardiovascular prognosis in obese patients is critical. In this regard, it is important to use indicators of obesity that are more strongly correlated with cardiovascular risk. Although BMI, as an overall obesity indicator, is the simplest and most widely used in clinical practice, it does not reflect muscle mass or fat distribution. Our findings, along with previous studies, showed that arterial stiffness, a good predictor of cardiovascular events^5,6^, was more frequently associated with indicators of abdominal obesity than BMI. Considering that the measurement of VFA requires specific equipment and technology, it is recommended to use WHR rather than of BMI as an indicator of obesity in clinical practice. WC is another indicator of abdominal obesity; however, it may underestimate risk in smaller people. Indeed, in our findings, WC correlated more strongly with baPWV than BMI, but not with WHR or VFA.

Several studies have reported improvement in arterial stiffness with weight loss^37^ and decreased VFA^38^. Other studies have found improvement in various metabolic factors and endothelial cell function in patients with abdominal obesity following therapeutic lifestyle intervention^39,40^. This indicates that it can improve arterial stiffness by controlling abdominal obesity. Based on the data so far, perhaps the best way to improve both arterial stiffness and abdominal obesity is aerobic exercise^41,42^. However, further research is needed to determine whether arterial stiffness improved by control of abdominal obesity leads to reduced cardiovascular risk.

We showed that the association between visceral adiposity and baPWV was more pronounced in younger subjects than older ones (Supplementary Table S2). Although underlying pathophysiology to explain this age-related difference is not clear, it is likely that other risk factors influencing arterial stiffness may play a stronger role with age. From a clinical point of view, this may mean that more aggressive management of abdominal obesity in young subjects is needed to reduce cardiovascular risk. However, more research results are needed to support our findings.

### Study limitations

This study has several limitations. First, as this is a cross-sectional study, the causal relationship between obesity and arterial stiffness could not be established. Second, data on concomitant medications were not available in our study. There was a possibility that some vasoactive medications had an influence on arterial stiffness. Lastly, the study population was restricted to Korean adults, so direct application of our results to other ethnic groups is difficult.

## Conclusions

Our study showed that baPWV was significantly correlated with WHR and VFA, but not with BMI. This implies that arterial stiffness may be more strongly associated with abdominal obesity than with overall obesity. Further studies with a larger sample size are needed to confirm our findings.

## Supplementary Information

Supplementary Information.

## Data Availability

All data generated or analyzed during this study are included in this article.

## Abbreviations

BMI: Body mass index
baPWV: Brachial-ankle pulse wave velocity
CI: Confidence interval
IRB: Institutional Review Board
OR: Odds ratio
WC: Waist circumference
WHR: Waist–hip ratio
VFA: Visceral fat area
