# Supplementary material for: Association between body fat parameters and arterial stiffness
Source: Sci Rep. 2021 Oct 15;11:20536. doi: 10.1038/s41598-021-00175-z (PMC8519992; doi:10.1038/s41598-021-00175-z)
Supplement: Supplementary file 1 — Supplementary Information. [file 41598_2021_175_MOESM1_ESM.docx]

**Supplementary data**

**Supplementary Figure S1. Correlation between VFA measured by VFA measured by INbody 720 and by computed tomography**


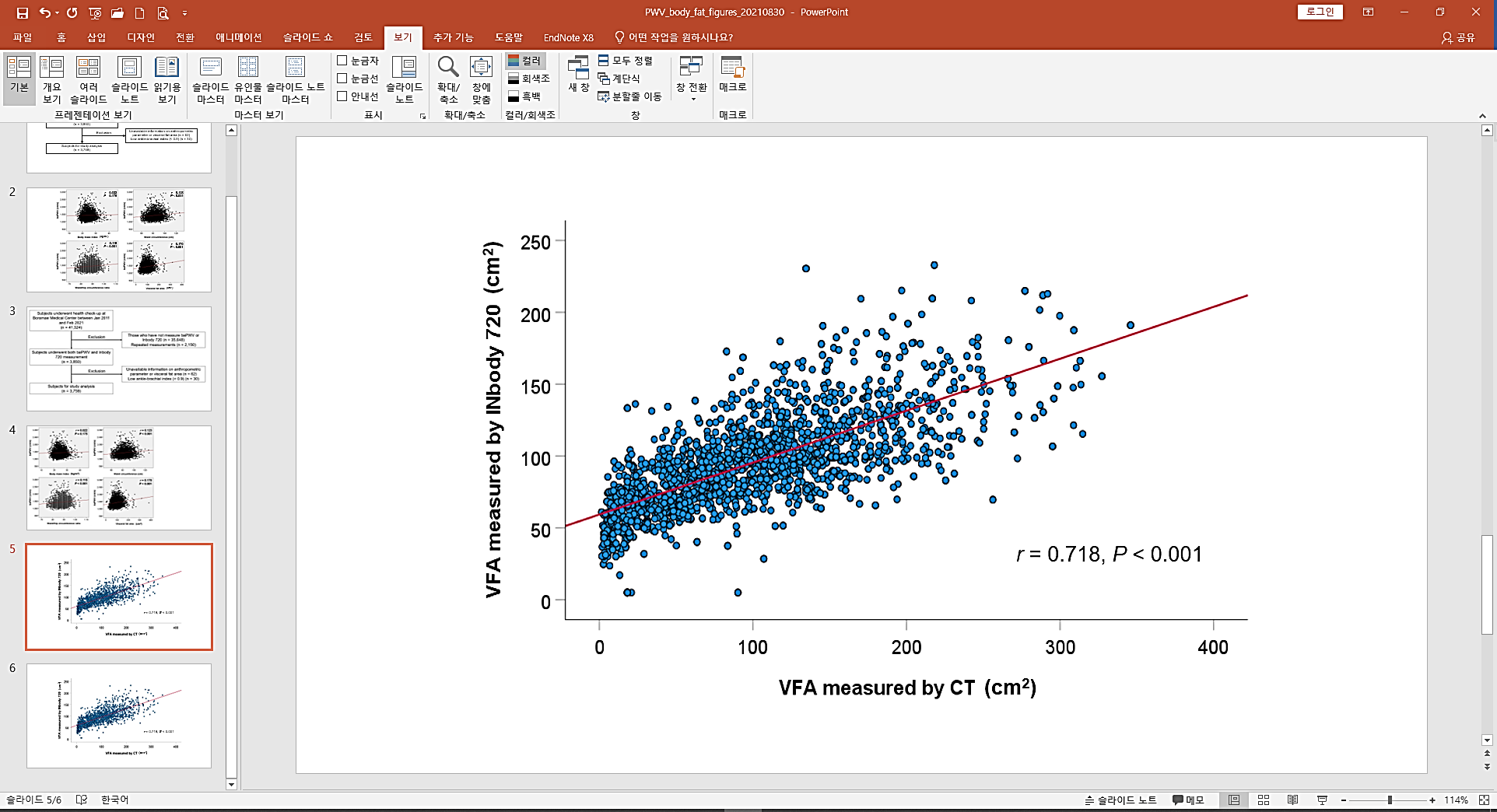


**Supplementary Table S1. Multiple binary logistic regression analyses showing independent associations of body fat parameters with higher baPWV* according to age**

| **Parameter** | **Age < 55 years (n = 2,076)** | |  | **Age ≥ 55 years (n = 1,682)** | |
| --- | --- | --- | --- | --- | --- |
|  | **OR (95% CI)** | ***P*** |  | **OR (95% CI)** | ***P*** |
| Body mass index ≥ 25 kg/m^2^ | 0.99 (0.75-1.09) | 0.319 |  | 0.92 (0.75-1.14) | 0.469 |
| Waist circumference, men ≥ 90 cm, women ≥ 85 cm | 0.95 (0.77-1.17) | 0.665 |  | 1.24 (0.99-1.56) | 0.051 |
| Waist-hip ratio, men > 0.90, women > 0.85 | 1.21 (1.01-1.46) | 0.046 |  | 1.23 (0.99-1.52) | 0.059 |
| Visceral fat area ≥ 100 cm^2^ | 1.43 (1.18-1.74) | < 0.001 |  | 1.16 (0.94-1.42) | 0.154 |

Following clinical covariates are controlled in each multivariable analysis: age, sex, hypertension, diabetes mellitus, dyslipidemia, cigarette smoking and alcohol drinking. *determined by median value of baPWV: ≥ 1,311 cm/s for those < 55 years and ≥1,470 cm/s for those ≥ 55 years. baPWV, brachial-ankle pulse wave velocity; OR, odds ratio; CI, confidence interval.

**Supplementary Table S2. Multiple linear regression analyses showing independent associations of body fat parameters with baPWV**

| **Parameter** | ***β**** | ***P*** |
| --- | --- | --- |
| Body mass index | 0.012 | 0.412 |
| Waist circumference | 0.085 | < 0.001 |
| Waist-hip ratio | 0.081 | < 0.001 |
| Visceral fat area | 0.116 | < 0.001 |

*Standardized coefficients. Age and sex are controlled in each multivariable analysis. baPWV, brachial-ankle pulse wave velocity; CI, confidence interval.

**Supplementary Table S3. Summary of studies showing the association between parameters of body fat arterial stiffness**

| **Source (year)** | **Study population** | **Number of subjects** | **Measure of body fat** | **Measure of arterial stiffness** | **Main result** | **Summary of findings** |
| --- | --- | --- | --- | --- | --- | --- |
| Wildman *et al*. (2003)^1^ | Healthy subjects | 336 | Body weight, BMI, WC, HC, WHR | aPWV | Positive association | All body fat parameters were strongly correlated with higher aPWV, independent of age, systolic blood pressure, race, and sex overall and among both age groups (*P* < 0.01 for all). |
| Rodrigues *et al*. (2012)^2^ | Community subjects | 1,608 | BMI, WC | cfPWV | No association | After controlling for blood pressure, age, heart rate and glucose, both BMI and WC were not associated with cfPWV (*P* > 0.05 for each). |
| Tang *et al*. (2020)^3^ | Healthy subjects | 578 | BMI | baPWV | Negative association | In multivariable analysis, BMI was negatively associated with baPWV (*β* = -0.06, *P* = 0.042). |
| Rider *et al*. (2010)^4^ | Obese subjects without other CVD risk factors | 50 | BMI, VFM | aPWV measured by MRI | Positive association (only with BMI) | Obesity was associated with a 14% increase in PWV (*P* = 0.021), and weight loss (average 50% excess weight) was associated with a 14% improvement in PWV (*P* = 0.03). VFM was not associated with aPWV. |
| Strasser *et al*. (2015)^5^ | Community subjects with CVD | 146 | BMI, WC | cfPWV, baPWV, VFM | Positive association | cfPWV and baPWV were associated with both BMI and WC even after controlling for age and sex (*P* < 0.05 for all). |
| Zhang et al. (2017)^6^ | Subjects underwent health check-up | 11,061 | BMI, WC, WHR, ABSI, BRI | baPWV | Positive association (only with WHR, ABSI and BRI) | Multivariable analysis showed that WHR, ABSI and BRI were associated with baPWV (*P* < 0.001). BMI was not associated with baPWV and WC was associated with WC in women (*P* < 0.05) but not in men (*P* > 0.005). |
| Heleniak *et al*. (2020)^7^ | Renal transplants recipients | 344 | BMI, WHR, BFM, FFM, PBF, TSFA, VFA | cfPWV, baPWV | No association | After adjustment for age and renal function, all body fat parameters were not associated with PWV (*P* < 0.05 for all). |
| Nordstrand *et al*. (2011)^8^ | Obese patients | 133 | WC, WHR, BMI, VFA | cfPWV | Positive association (only in women) | Multiple linear regression analysis showed that increasing BMI, WC, WHR, VFA and BFM were independently associated with higher cfPWV in women, but not in men, after adjustment for age, hypertension and type 2 diabetes (*P* < 0.05 for all). |
| Choi *et al*. (2019)^9^ | Subjects underwent health check-up | 2,647 | BMI, WC, ABSI, BRI, VAI | baPWV | Positive association (only with ABSI and VAI) | In the multivariate regression analysis, ABSI  and VAI were found to be significantly correlated with the mean baPWV in both men and women (*P* < 0.05 for all). |

BMI, body mass index; WC, waist circumference; HC, hip circumference; WHR, waist-hip ratio; aPWV, aortic pulse wave velocity; cfPWV, carotid-femoral pulse wave velocity; baPWV, brachial-ankle pulse wave velocity; CVD cardiovascular disease; aPWV, aortic pulse wave velocity; VFM, visceral fat mass; MRI, magnetic resonance imaging; ABSI, body shape index; BRI, body roundness index; BFM, body fat mass; FFM, fat free mass; PBF, percent body fat; TSFA, trunk segmental fat analysis; VAI, visceral adiposity index.

**References**

1 Wildman, R. P., Mackey, R. H., Bostom, A., Thompson, T. & Sutton-Tyrrell, K. Measures of obesity are associated with vascular stiffness in young and older adults. *Hypertension* **42**, 468-473, doi:10.1161/01.hyp.0000090360.78539.cd (2003).

2 Rodrigues, S. L. *et al.* Body mass index is not independently associated with increased aortic stiffness in a Brazilian population. *Am J Hypertens* **25**, 1064-1069, doi:10.1038/ajh.2012.91 (2012).

3 Tang, B. *et al.* Relationship between body mass index and arterial stiffness in a health assessment Chinese population. *Medicine (Baltimore)* **99**, e18793 (2020).

4 Rider, O. J. *et al.* The effect of obesity and weight loss on aortic pulse wave velocity as assessed by magnetic resonance imaging. *Obesity (Silver Spring)* **18**, 2311-2316, doi:10.1038/oby.2010.64 (2010).

5 Strasser, B. *et al.* Abdominal obesity is associated with arterial stiffness in middle-aged adults. *Nutr Metab Cardiovasc Dis* **25**, 495-502, doi:10.1016/j.numecd.2015.01.002 (2015).

6 Zhang, J. *et al.* Comparison of the ability to identify arterial stiffness between two new anthropometric indices and classical obesity indices in Chinese adults. *Atherosclerosis* **263**, 263-271, doi:10.1016/j.atherosclerosis.2017.06.031 (2017).

7 Heleniak, Z. *et al.* Obesity, Fat Tissue Parameters, and Arterial Stiffness in Renal Transplant Recipients. *Transplant Proc* **52**, 2341-2346, doi:10.1016/j.transproceed.2020.01.118 (2020).

8 Nordstrand, N. *et al.* The relationship between various measures of obesity and arterial stiffness in morbidly obese patients. *BMC Cardiovasc Disord* **11**, 7 (2011).

9 Choi, H. S. *et al.* Association between new anthropometric parameters and arterial stiffness based on brachial-ankle pulse wave velocity. *Diabetes Metab Syndr Obes* **12**, 1727-1733 (2019).
